# Risk factors for intimate partner violence and abuse among adolescents and young adults: findings from a UK population-based cohort

**DOI:** 10.12688/wellcomeopenres.16106.3

**Published:** 2021-01-21

**Authors:** Annie Herbert, Jon Heron, Christine Barter, Eszter Szilassy, Maria Barnes, Laura D. Howe, Gene Feder, Abigail Fraser

**Affiliations:** 1 Department of Population Health Sciences, University of Bristol, Bristol, UK; 2 MRC Integrative Epidemiology Unit, University of Bristol, Bristol, UK; 3 University of Central Lancashire, Preston, UK; 4 Centre for Academic Primary Care, University of Bristol, Bristol, UK

**Keywords:** ALSPAC, Intimate Partner Violence, Dating Violence, Adolescent, Young Adult, Cohort Studies

## Abstract

**Background:** Approximately one-third of young people in the UK have suffered intimate partner violence and abuse (IPVA) on reaching adulthood. We need interventions to prevent IPVA in this population, but there is a lack of evidence on who is at greatest risk.

**Methods:** We analysed questionnaire data from 3,279 participants of the Avon Longitudinal Study of Parents and Children population-based birth cohort. We estimated the prevalence of IPVA victimisation and perpetration by age 21, by sex, demographic, parenting, mental health, externalising behaviour (e.g. smoking), educational, employment, and adverse childhood factors.

**Results:** Overall, 29% of males and 41% of females reported IPVA victimisation, with 20% and 25% reporting perpetration, respectively (16% and 22% both). The most common type of IPVA was emotional, followed by physical, then sexual. History of anxiety, self-harm, anti-social behaviour, cannabis or illicit (non-cannabis) drug use, or risky sexual behaviour among males and females were associated with a 50% increase in likelihood of IPVA (victimisation or perpetration). Males reporting depression, sexual abuse (not by an intimate partner), witnessing domestic violence, or parental separation were also more likely to experience IPVA. Extreme parental monitoring, high academic achievement during adolescence, and NEET (not being in education, employment, or training) status in young adulthood were associated with reduced risks of IPVA.

**Conclusions:** A range of demographic, mental health, and behavioural factors were associated with increased prevalence of IPVA victimisation or perpetration. Further study of likely complex pathways from these factors to IPVA, to inform primary prevention, is needed.

## Introduction

Intimate partner violence and abuse (IPVA), defined as the physical, emotional/psychological, or sexual abuse by a current or former partner, is associated with acute, short-term effects such as injury, and poor long term physical and mental health, such as obesity and depression ^1, 2^, as well as substantial social and economic costs ^3^. Public health approaches that can support individuals at high risk for IPVA, to prevent its occurrence or mitigate its adverse effects are needed, and have increasingly become a focus of the UK government ^4^. Adolescence and young adulthood is a time when most individuals establish their beliefs around peer and dating relationships ^5^, and as such, may be an ideal phase in the life course to identify high-risk individuals for primary prevention ^6^.

According to data from the Avon Longitudinal Study of Parents and Children (ALSPAC), a birth cohort established in the early 1990s, approximately 37% of young people in the UK have been exposed to IPVA victimisation by the time they are 21 years old ^7^. However, which groups of young people in the UK might be at greatest risk of IPVA victimisation or perpetration, and might most benefit from intervention, is currently not well understood ^2, 8^. Most evidence on risk or protective factors come from studies of North American populations, which are likely to differ both culturally and in terms of educational, social, health, and judicial systems, compared to young people in other countries ^8– 14^. Further, most studies of IPVA have been in small or unrepresentative samples, are of adolescents aged under 18 or older adults, are in young girls and women only, or investigate risk or protective factors for victimisation but not perpetration ^8– 14^. There is a clear need for contemporary information from large studies, and for a deeper understanding of pathways to IPVA with which to inform the development and evaluation of prevention strategies.

We therefore investigated risk factors for IPVA occurring up to age 21 in a large UK population-based birth cohort. The aim was to identify subgroups of adolescents and young adults who are at greatest risk of either IPVA victimisation or perpetration.

## Methods

### Data

We conducted a cross-sectional analysis of data from birth to 21 years old on participants from the ALSPAC cohort. ALSPAC recruited ~14,500 pregnant women residing in Avon, UK, with expected delivery dates in April 1991–December 1992 (approximately three-quarters of the eligible population) and has collected information on the mothers, partners, and their offspring, on a wide range of mental, physical, economic, and social factors, for the subsequent 25 years. Study data were collected and managed using REDCap electronic data capture tools hosted at University of Bristol ^15^. More information on ALSPAC is available within published cohort profiles ^16– 18^. The study website contains details of all the data that is available through a fully searchable data dictionary and variable search tool ^19^.

We focussed on participants in the age 21 wave (median and interquartile range [IQR] age 21, 21 to 22). All eligible participants who could be contacted (n=9,353) were provided details of an online questionnaire in mid-December 2013, and then sent a series of up to four reminders at three-week intervals, some of these reminders containing a paper version of the same questionnaire. The dataset for the age 21 wave consisted of 3,459/9,353 (37%) who had responded. The current study’s cohort was the 3,279 who answered questions within the IPVA section (minus one participant where sex was missing). Data were not available on reasons for non-contact or non-response.

### Characteristics of study cohort

Characteristics of the 1,149 males and 2,130 females in the study cohort have been reported elsewhere ^7^. In summary, the majority of participants were white, lived with both parents, had a mother that was married, and had parents who were both in professional, managerial or skilled occupations when they were born. By age 16, approximately two-thirds defined themselves as *‘100% heterosexual’* (as opposed to *‘mostly heterosexual’*, *‘bisexual’*, etc.; noting that over one-quarter of data on sexual orientation was missing), around half had reported having had at least one ACE (adverse childhood experience), and around one-fifth of girls reported having self-harmed ( *Extended data*, Table C) ^20^. By age 18, around one-fifth of girls and boys reported drinking hazardous levels of alcohol or risky sexual behaviour, such as not using contraception, and one in ten had ever been hospitalised.

As, by definition, IPVA occurs within intimate relationships, we estimated how many of the study cohort had been in a relationship by age 21, through two questions explicitly capturing this at ages 13 and 17, and augmented by responses to other questions at ages 12–21 (described in more detail in *Extended data*, Table A) ^20^. This indicated minimum prevalence of relationships that were still likely under-captured, so we did not restrict the analysis according to these questions. Over half of the study cohort explicitly said that they had been in a relationship by age 18 (57%), and 74% indicated this, increasing to 88% by the time they were 21. These proportions were similar between men and women. Young people were less likely to report being in a relationship by age 21 if they were non-White (non-White vs. White men: 71% vs. 85%, women: 75% vs. 90%), with little difference between those of different ‘deprivation’ (socio-economic) categories or sexual orientation.

### Exposures

We investigated individual, relational, and community characteristics, as potential risk factors, based on previous literature ^8– 14^. These factors were: high area-level deprivation (i.e. being resident in a geographical area with a high ‘Index of Multiple Deprivation’ score at age 21 ^21^; an indicator of socio-economic status), ethnic minority status (birth records, augmented by data at later waves), sexual minority status (ages 15 and 21), history of: depression (ages 16 and 18), anxiety (ages 15 and 17), self-harm (ages 16 and 17), anti-social behaviours (ages 13 and 18), substance misuse (smoking, cannabis use, regular illicit [non-cannabis] drug use – ages 16 and 18; hazardous alcohol use at age 18), risky sexual behaviours (age 12–17), high levels of parental monitoring (age 15), hospitalisations (age 15–18), low educational attainment (age 13–14 and 16), and Not in Employment, Education or Training (NEET) status (ages 18 and 20). We also investigated 11 different types of adverse childhood experiences (ACEs, e.g. witnessing domestic violence, by age 16) ^22^. Further details on how these variables were derived are provided in *Extended data*, Table B ^20^.

For most exposures, we imputed any missing values using multiple imputation via chained equations. We assumed values to be missing at random and sufficient auxiliary information with which to impute, except for ethnicity, sexual orientation, risky sexual behaviour, and hospitalisation. We also included imputed ACE variables as previously described ^22^. Further details on imputation methods used are provided in *Extended data*, Box A ^20^.

### Outcome: IPVA

The IPVA section of the questionnaire at age 21 was based on previous UK and European questionnaires and the PROVIDE questionnaire ^23, 24^, and is described in full in a paper validating its psychometric properties ^7^. Questions asked about occurrence of eight different examples of emotional, physical, and sexual IPVA victimisation within intimate relationships, including one-night stands (e.g.: *‘Used physical force such as pushing, slapping, hitting or holding you down?’* – physical victimisation). The questionnaire did not distinguish between the length/type of relationship (e.g. long-term, one-night stand), as in previous research from our group, relationships for young people (and the young people’s own understandings/interpretations of what it means being in a relationship) are more difficult to categorise than for older adults ^23, 25^. Participants were also asked the frequency of these events ( *‘never’*, *‘once’*, *‘a few times’*, ‘ *often’*), and whether they occurred before/after turning 18 or both periods. There were also four similarly worded (but more condensed) questions on occurrence and frequency of emotional, physical, and sexual IPVA perpetration. Participants were also asked ‘How did you feel after they did these things to you?’ following the batch of victimisation questions, with ten different response options (seven negative impacts, e.g. *‘upset/unhappy’*, one neutral – *‘no effect/not bothered’*, two positive, e.g. *‘felt loved/protected/wanted’*).

For the purpose of this study, we considered a participant to have experienced IPVA victimisation, perpetration, and their different types (e.g. emotional), if they had responded at least *‘once’* for any of the respective questions. It has been previously argued that thresholds should be carefully considered for certain types such as emotional victimisation to avoid over-estimating IPVA ^26^. We defined the cut-off ‘never’ vs. ‘ever’, for two reasons. Firstly, the header of the questionnaire was *‘Intimate Partner Violence’*, likely raising the threshold of severity for reporting certain behaviours. Secondly, for participants who answered ‘ever’ to any of the eight different victimisation questions, i.e. including those relating to emotional IPVA, negative impact was reported by 75–99%.

### Statistical analyses

We estimated the prevalence of IPVA victimisation and perpetration, respectively, up to age 17, at age 18–21, and ‘ever’ (by age 21), overall and by each factor of interest (i.e. IPVA victimisation and perpetration were handled as binary variables; exposure variables categorical). Among those reporting any IPVA victimisation, we reported the proportions who reported negative, neutral, or positive impact. We then presented the association between each of the factors of interest with ‘ever’ IPVA victimisation and perpetration, respectively, as risk ratios and 95% confidence intervals. These risk ratios were approximated from estimated odds ratios as per Zhang *et al.* ^27^

We stratified all analyses by sex (recorded at birth), given that a large part of the literature focuses on violence against girls and women ^9, 12, 28^, and to allow comparison with previous reports. There were insufficient data to also incorporate gender (identity) in analyses. Again, for comparability with previous work, we present prevalence of IPVA types, i.e. emotional, physical, and sexual IPVA, respectively.

In the main text of this report, we present results on exposures where missing values have been imputed; the same results for completely observed exposures are presented in *Extended data*, Table F (noting that sample sizes will vary between exposures) ^20^.

We analysed all data in Stata version 15.1, except for multiple imputation, which was carried out in R version 3.5.3. As per disclosure rules for use of ALSPAC data, we do not report any numbers (or related percentages) less than 5. The R script used for analyses is available at: https://github.com/pachucasunrise/RFs_IPVA.

## Consent and ethical approval

Written informed consent was obtained from the parents of participating children after receiving a full explanation of the study. Children were invited to give assent where appropriate. Study members have the right to withdraw their consent for elements of the study or from the study entirely at any time. Full details of the ALSPAC consent procedures are available on the study website. The questions on IPVA were approved by the ALSPAC Ethics and Law Committee (ref: E201210).

## Results

### Prevalence of IPVA victimisation and perpetration

Overall, 29% of males and 41% of females reported ever being victimised, and 20% and 25% reported ever perpetrating IPVA ( Table 1); 16% and 22% reported both victimisation and perpetration. Emotional IPVA was the most common type, present in the majority of victimisation and perpetration reports; 14% and 17% of men and women, respectively, reported experiencing either emotional victimisation or emotional perpetration but no other type. IPVA victimisation and perpetration were more likely at an older age ( Table 1): 263 (8%) reported being victimised both before and after turning 18 years old (men: 6%; women: 9%), and 130 reported perpetrating during both periods (men: 3%, women: 4%). Rates of reported physical perpetration were higher in women than in men (9% vs. 2%) ( Table 1). Sexual IPVA perpetration was reported by 2% of men and 0.3% of women.

**Table 1.** Prevalence of victimisation and perpetration types by sex and age at when it was reported ^a^.

	Men (n=1,149)						Women (n=2,130)					
	0–17y		18–21y		0–21y		0–17y		18–21y		0–21y	
Victimisation												
Any	110	(9.6)	275	(23.9)	330	(28.7)	377	(17.7)	683	(32.1)	880	(41.3)
Emotional	98	(8.5)	247	(21.5)	300	(26.1)	295	(13.8)	572	(26.9)	753	(35.4)
Physical	35	(3)	89	(7.7)	115	(10)	145	(6.8)	268	(12.6)	379	(17.8)
Sexual	12	(1)	45	(3.9)	54	(4.7)	191	(9.0)	252	(11.8)	388	(18.2)
Perpetration												
Any	72	(6.3)	181	(15.8)	227	(19.8)	154	(7.2)	442	(20.8)	539	(25.3)
Emotional	68	(5.9)	169	(14.7)	215	(18.7)	140	(6.6)	406	(19.1)	501	(23.5)
Physical	8	(0.7)	20	(1.7)	28	(2.4)	57	(2.7)	150	(7.0)	200	(9.4)
Sexual	8	(0.7)	15	(1.3)	23	(2.0)	5	(0.2)	<5	(<0.2)	7	(0.3)

^a^. Data shown are ns (% of 1,149 men or 2,130 women). Counts <5 not displayed to avoid disclosure.

### Impact of IPVA victimisation

Prevalence of victimisation and perpetration by all factors studied and age (up to 17 years old, between 18 and 21, and at any age up to 21), are presented in *Extended data*, Tables D–E.

**Figure 1.**
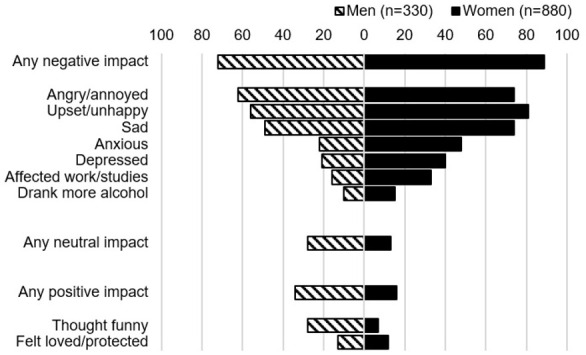
Prevalence (%) of different types of impact among participants who reported any victimisation.

Over 60% who reported any IPVA victimisation also reported experiencing a negative impact, the most likely impacts were feeling angry/annoyed, upset/unhappy, or sad ( Figure 1). Women were more likely than men to report each of the seven negative types, and men were more likely to report any neutral or positive impacts.

### Risk factors for victimisation

According to point estimates, nearly all factors studied were positively associated with IPVA victimisation by age 21, except for high levels of parental monitoring at age 15, relatively high academic achievement (Key Stage 3 scores higher than 117 at age 13-14 or five or more A*-C GCSE grades at age 16), and NEET status at age 20, which were negatively associated ( Table 2). Risks of victimisation were also increased if reporting ACEs by age 16 for most types, except emotional neglect for either sex, bullying for men, or witnessing violence between parents for women, but these estimates were imprecise ( Table 3).

**Table 2.** Relative risks of intimate partner violence and abuse (IPVA) by 21 years old by socio-demographic/clinical variables and sex ^a^.

	Outcome: Victimisation						Outcome: Perpetration					
	Men (n=1,149)			Women (n=2,130)			Men (n=1,149)			Women (n=2,130)		
Variable (age that variable covers)	%	RR (95% CI)		%	RR (95% CI)		%	RR (95% CI)		%	RR (95% CI)	
**Demographics**												
Deprivation level (21y)												
1 – Lowest level of deprivation	28.5	(ref)		38.6	(ref)		19.2	(ref)		21.7	(ref)	
2	30.3	1.06	(0.81, 1.36)	41.4	1.05	(0.83, 1.29)	18.8	0.98	(0.68, 1.37)	27.2	0.98	(0.69, 1.36)
3	24.9	0.88	(0.62, 1.19)	40.2	0.89	(0.66, 1.16)	17.0	0.88	(0.58, 1.3)	24.1	0.89	(0.59, 1.29)
4	28.8	1.01	(0.73, 1.35)	44.4	1.01	(0.76, 1.29)	23.3	1.21	(0.82, 1.72)	26.6	1.21	(0.83, 1.68)
5 – Highest level of deprivation	33.1	1.16	(0.79, 1.6)	45.2	1.14	(0.82, 1.48)	24.8	1.29	(0.81, 1.93)	30.5	1.28	(0.81, 1.87)
Ethnicity (birth ^b^)												
White	28.5	(ref)		41.3	(ref)		19.7	(ref)		25.2	(ref)	
Non-White	38.1	1.28	(0.88, 1.69)	38.9	1.27	(0.88, 1.67)	16.7	0.84	(0.40, 1.63)	26.7	0.86	(0.43, 1.51)
Sexual orientation (15.5y)												
100% heterosexual	26.2	(ref)		38.8	(ref)		18.7	(ref)		22.0	(ref)	
Not 100% heterosexual	40.4	1.54	(1.15, 1.97)	55.1	1.41	(1.12, 1.69)	23.6	1.26	(0.83, 1.83)	37.2	1.25	(0.83, 1.77)
**Internalising** **symptoms/** **behaviours**												
Depression symptoms in past two weeks												
At 16y												
No	27.3	(ref)		38.2	(ref)		18.6	(ref)		22.9	(ref)	
Yes	39.2	1.44	(1.11, 1.79)	51.0	1.35	(1.10, 1.6)	28.1	1.51	(1.08, 2.02)	32.6	1.47	(1.08, 1.92)
At 18y												
No	27.3	(ref)		37.8	(ref)		18.6	(ref)		22.9	(ref)	
Yes	35.6	1.31	(1.00, 1.65)	49.6	1.25	(1.00, 1.51)	25.4	1.36	(0.98, 1.83)	31.1	1.34	(0.98, 1.76)
Anxiety symptoms												
At 15.5y (in past six months)												
No	28.5	(ref)		40.8	(ref)		19.7	(ref)		24.8	(ref)	
Yes	42.9	1.49	(0.58, 2.57)	54.7	1.37	(0.63, 2.03)	19.3	0.92	(0.20, 2.79)	38.5	0.93	(0.21, 2.51)
At 17.5y												
No	27.7	(ref)		39.4	(ref)		18.9	(ref)		23.4	(ref)	
Yes	43.5	1.56	(1.09, 2.07)	53.8	1.43	(1.07, 1.76)	32.4	1.70	(1.06, 2.49)	37.3	1.63	(1.05, 2.30)
Self-harm behaviours ever												
At 16y												
No	26.7	(ref)		35.5	(ref)		18.1	(ref)		21.1	(ref)	
Yes	44.6	1.66	(1.30, 2.04)	57.4	1.54	(1.25, 1.82)	32.7	1.80	(1.31, 2.37)	36.8	1.75	(1.29, 2.26)
At 17.5y												
No	27.2	(ref)		36.8	(ref)		18.9	(ref)		21.9	(ref)	
Yes	41.0	1.50	(1.14, 1.89)	56.6	1.41	(1.12, 1.69)	26.8	1.42	(0.98, 1.97)	36.6	1.40	(0.98, 1.90)
**Externalising** **behaviours**												
Anti-social behaviour in past 12 months ^c^												
At 13y												
No/not reported ^d^	28.1	(ref)		40.5	(ref)		18.7	(ref)		24.0	(ref)	
Yes	34.7	1.24	(0.89, 1.63)	50.0	1.19	(0.91, 1.47)	30.1	1.60	(1.12, 2.19)	38.8	1.54	(1.11, 2.03)
At 18y												
No/not reported ^d^	27.6	(ref)		40.4	(ref)		18.7	(ref)		24.5	(ref)	
Yes	38.7	1.40	(1.01, 1.83)	57.1	1.31	(1.01, 1.60)	29.4	1.56	(1.04, 2.20)	39.9	1.50	(1.04, 2.02)
Current cigarette smoking, at least weekly												
At 16y												
No	28.0	(ref)		39.3	(ref)		19.4	(ref)		23.9	(ref)	
Yes	37.1	1.32	(0.93, 1.77)	55.3	1.26	(0.94, 1.58)	24.3	1.25	(0.77, 1.89)	35.3	1.23	(0.78, 1.81)
At 18y												
No	27.0	(ref)		38.4	(ref)		18.4	(ref)		23.5	(ref)	
Yes	38.3	1.42	(1.06, 1.81)	56.7	1.33	(1.05, 1.60)	27.7	1.49	(1.06, 2.03)	34.6	1.45	(1.05, 1.90)
Past year hazardous alcohol use at 18y												
No	28.1	(ref)		40.6	(ref)		17.1	(ref)		23.3	(ref)	
Yes	29.6	1.05	(0.87, 1.25)	42.2	1.04	(0.89, 1.20)	23.3	1.36	(1.08, 1.69)	27.7	1.33	(1.07, 1.61)
Current/past month cannabis use, at least weekly												
At 16y												
No	28.0	(ref)		40.8	(ref)		19.0	(ref)		24.7	(ref)	
Yes	42.3	1.50	(0.99, 2.07)	67.0	1.38	(0.99, 1.74)	33.9	1.77	(1.07, 2.64)	54.8	1.68	(1.06, 2.37)
At 18y												
No	27.6	(ref)		40.4	(ref)		18.9	(ref)		24.6	(ref)	
Yes	38.4	1.39	(1.00, 1.82)	62.2	1.30	(1.00, 1.59)	27.5	1.44	(0.95, 2.08)	41.4	1.40	(0.95, 1.93)
Any current/past month illicit (non-cannabis) drug use												
At 16y												
No	27.9	(ref)		40.1	(ref)		19.0	(ref)		24.2	(ref)	
Yes	43.0	1.53	(1.02, 2.09)	60.2	1.41	(1.02, 1.77)	33.7	1.76	(1.09, 2.58)	43.4	1.68	(1.08, 2.35)
At 18y												
No	27.7	(ref)		39.8	(ref)		18.6	(ref)		24.4	(ref)	
Yes	36.9	1.33	(0.98, 1.73)	59.2	1.26	(0.98, 1.54)	29.4	1.57	(1.08, 2.17)	36.2	1.51	(1.08, 2.00)
Risky sexual behaviour (at 12.5–17.5y)												
No/not reported ^d^	24.2	(ref)		37.4	(ref)		17.4	(ref)		21.6	(ref)	
Yes	45.3	1.87	(1.58, 2.17)	51.3	1.62	(1.43, 1.80)	28.6	1.65	(1.29, 2.05)	34.9	1.59	(1.27, 1.95)
**Relationships/** **experiences**												
Current parental monitoring levels (15.5y)												
Low/average	30.4	(ref)		47.3	(ref)		21.3	(ref)		30.6	(ref)	
High	26.0	0.85	(0.69, 1.05)	33.3	0.89	(0.74, 1.04)	17.2	0.81	(0.60, 1.07)	18.1	0.83	(0.63, 1.06)
Hospitalisations (at 15.5–18y)												
No/not reported ^d^	28.6	(ref)		40.1	(ref)		19.8	(ref)		24.5	(ref)	
Yes	29.4	1.03	(0.77, 1.33)	50.4	1.02	(0.8, 1.27)	19.1	0.96	(0.66, 1.37)	31.5	0.97	(0.67, 1.34)
**Education/training**												
Key Stage 3 scores (at age 13–14y)												
Key Stage 3 score < 117	30.0	(ref)		45.7	(ref)		19.6	(ref)		27.6	(ref)	
Key Stage 3 score >= 117	28.0	0.93	(0.74, 1.15)	38.4	0.95	(0.79, 1.11)	19.9	1.01	(0.76, 1.32)	23.7	1.01	(0.78, 1.28)
GCSE grades (at age 16y)												
< 5 A*-C GCSE grades	31.6	(ref)		47.8	(ref)		18.3	(ref)		29.6	(ref)	
>= 5 A*-C GCSE grades	28.1	0.89	(0.68, 1.14)	40.2	0.92	(0.74, 1.10)	20.0	1.10	(0.76, 1.54)	24.6	1.08	(0.78, 1.43)
NEET status												
At age 18y												
No	28.1	(ref)		41.0	(ref)		19.4	(ref)		25.1	(ref)	
Yes	36.0	1.27	(0.85, 1.77)	47.3	1.21	(0.87, 1.56)	24.4	1.25	(0.75, 1.93)	28.7	1.23	(0.76, 1.81)
At 20y												
No	36.0	(ref)		47.3	(ref)		24.4	(ref)		28.7	(ref)	
Yes	29.4	0.67	(0.36, 1.14)	40.8	0.71	(0.40, 1.12)	20.2	0.69	(0.33, 1.31)	24.8	0.70	(0.35, 1.29)

CI = Confidence Interval; GCSE = General Certificate of Secondary Education; NEET = Not in Employment, Education, or Training; RR = Relative Risk a. Missing risk factor data were imputed; %s and RRs represent pooled results. For further details, see *Extended data*, Box A ^20^. b. Augmented by school census responses at 9–13 years old. c. Not including activities that also come under the definition for IPVA (e.g *. ‘really hurt someone or been physically cruel to them (e.g. has tied up, cut or burned someone)’*. See *Extended data*, Table B ^20^. d. *‘No/not reported’* means that the participant’s response was *‘no’* and/or missing for all these categories.

**Table 3.** Relative risks of intimate partner violence and abuse (IPVA) by 21 years old by adverse childhood experiences (ACEs) and sex.

		Outcome: Victimisation						Outcome: Perpetration					
ACE variable (age that variable covers)		Men (n=1,149)			Women (n=2,130)			Men (n=1,149)			Women (n=2,130)		
		%	RR	(95% CI)	%	RR	(95% CI)	%	RR	(95% CI)	%	RR	(95% CI)
Any ACE (0–16y)	No/ not reported ^a^	26.0	(ref)		36.1	(ref)		16.2	(ref)		21.2	(ref)	
	Yes	29.7	1.14	(0.92, 1.39)	41.8	1.03	(0.93, 1.14)	20.2	1.07	(0.84, 1.35)	26.4	1.10	(0.97, 1.23)
ACE type													
Emotional abuse (0–11y)	No	26.3	(ref)		39.7	(ref)		19.4	(ref)		24.1	(ref)	
	Yes	33.3	1.27	(1.00, 1.57)	45.6	1.20	(1.05, 1.36)	20.6	1.10	(0.82, 1.43)	29.9	1.23	(1.05, 1.40)
Physical abuse (0–11y)	No	25.6	(ref)		37.4	(ref)		16.9	(ref)		22.7	(ref)	
	Yes	37.8	1.47	(1.18, 1.79)	46.9	1.25	(1.09, 1.42)	28.2	1.56	(1.23, 1.91)	32.0	1.31	(1.13, 1.48)
Sexual abuse (0–16y)	No	27.5	(ref)		39.6	(ref)		18.9	(ref)		24.6	(ref)	
	Yes	50.0	1.82	(0.88, 2.76)	56.5	1.43	(1.16, 1.68)	33.3	1.63	(0.71, 2.66)	37.0	1.36	(1.09, 1.63)
Emotional neglect (0–16y)	No	29.2	(ref)		41.6	(ref)		21.1	(ref)		25.4	(ref)	
	Yes	26.0	0.89	(0.68, 1.14)	39.6	0.95	(0.81, 1.10)	14.4	0.71	(0.5, 0.97)	21.2	0.87	(0.70, 1.04)
Bullying (8–16y)	No	29.6	(ref)		39.5	(ref)		19.8	(ref)		23.5	(ref)	
	Yes	27.7	0.94	(0.74, 1.16)	48.4	1.22	(1.08, 1.37)	19.7	0.99	(0.77, 1.26)	32.3	1.27	(1.12, 1.43)
Witnessed domestic violence (0–12y)	No	26.5	(ref)		40.4	(ref)		17.5	(ref)		25.3	(ref)	
	Yes	36.0	1.36	(1.06, 1.70)	36.9	0.91	(0.76, 1.08)	31.3	1.65	(1.30, 2.02)	21.6	0.88	(0.71, 1.07)
Parental substance abuse (0–11y)	No	27.2	(ref)		38.5	(ref)		18.7	(ref)		23.8	(ref)	
	Yes	29.9	1.10	(0.75, 1.53)	48.5	1.26	(1.03, 1.49)	18.2	0.98	(0.61, 1.46)	30.3	1.21	(0.96, 1.46)
Parental mental illness or suicide attempt (0–16y)	No	24.6	(ref)		38.2	(ref)		16.7	(ref)		24.5	(ref)	
	Yes	32.6	1.33	(1.09, 1.59)	41.5	1.09	(0.96, 1.21)	22.5	1.30	(1.04, 1.60)	24.7	1.01	(0.87, 1.15)
Parent criminal conviction (0–12y)	No	27.1	(ref)		39.1	(ref)		18.7	(ref)		24.9	(ref)	
	Yes	36.5	1.35	(0.93, 1.83)	47.6	1.22	(0.97, 1.47)	20.6	1.09	(0.67, 1.63)	26.2	1.04	(0.78, 1.33)
Parental separation (0–16y)	No	24.6	(ref)		37.2	(ref)		17.6	(ref)		23.5	(ref)	
	Yes	37.4	1.52	(1.22, 1.85)	44.8	1.20	(1.05, 1.36)	23.6	1.30	(1.00, 1.66)	26.7	1.11	(0.94, 1.28)
Number of ACEs (0–16y)	0	26.0	(ref)		40.4	(ref)		18.6	(ref)		23.3	(ref)	
	1	25.3	0.97	(0.74, 1.24)	38.1	0.94	(0.81, 1.08)	17.0	0.92	(0.67, 1.22)	23.5	1.01	(0.86, 1.16)
	2	29.4	1.13	(0.85, 1.45)	41.8	1.03	(0.89, 1.19)	20.2	1.07	(0.77, 1.44)	28.0	1.15	(0.98, 1.32)
	3+	36.6	1.40	(1.10, 1.74)	46.7	1.15	(1.01, 1.30)	24.8	1.29	(0.97, 1.66)	28.6	1.17	(1.01, 1.34)

a. ‘No/not reported’ means that the participant’s response was ‘no’ and/or missing for all ACEs.

Risks of victimisation by age 21 were highest (at least 50% higher) for males and females if they reported having self-harmed at age 16 or had engaged in risky sexual behaviour by age 17 ( Table 2). They were also high for males who suffered anxiety symptoms at age 17, regularly used cannabis or other illicit drugs at age 16 ( Table 2), or who had been sexually abused (not by an intimate partner) or whose parents had separated by age 16 ( Table 3).

### Risk factors for perpetration

The risk of IPVA perpetration was also increased for nearly all factors studied, except for ethnic minority status, high levels of parental monitoring, or NEET status at age 20 ( Table 2; according to point estimates, noting that confidence intervals for negative associations tended to be wide and include unity). Point estimates for risks of perpetration were also increased for both men and women exposed to ACEs by age 16 for most categories ( Table 3).

Risks of perpetration by age 21 were highest (at least 50% higher) in men and women who reported engaging in anti-social behaviour at ages 13 or 18, anxiety symptoms at age 17, self-harm at age 16, regular cannabis use at age 16, illicit (non-cannabis) drug use at ages 16 or 18, or risky sexual behaviour at age 17 ( Table 2). Risks were also greater for men who suffered depression symptoms, or who had been sexually abused or had witnessed domestic violence by age 16 ( Table 3).

### Sensitivity analyses

Distributions of factors after imputing missing values tended towards greater adversity (i.e. higher proportions of an adverse factor, e.g. anxiety at age 18: 6% vs. 4% in observed data only), which is often the case, given that more vulnerable young people (socioeconomically and otherwise) are more likely to be missing from analyses ^29^. When we compared results using multiple imputation ( Table 2) with those in observed data only ( *Extended data*, Table F), findings were very similar. The median difference in model coefficients was 6% (IQR: 2% to 23%). The largest differences were for deprivation (level 3 vs. level 1) and its association with victimisation in men, and NEET status at age 18 (vs. no such status) and its association with perpetration in men (RRs in main analyses: 0.88 and 1.25, respectively; RR in observed data only: 0.85 and 1.10).

## Discussion

In a contemporary UK population-based cohort, almost three out of ten young men and more than four out of ten young women reported having been exposed to IPVA by the time they were 21, and one in five men and one in four women reported having perpetrated IPVA. We show that these risks were increased for men and women as they turned 18, particularly for those who reported self-harm, anti-social behaviour, regular cannabis, or illicit (non-cannabis) drug use by adolescence. Men who engaged in risky sexual behaviour, had been sexually abused (not by an intimate partner), or had witnessed domestic violence, and sexual minority women, were also at increased risks.

### Strengths & Limitations

This study was carried out in a population-based cohort, with a rich range of individual, relational and community-level variables of interest. We used a validated scale to capture IPVA victimisation ^7^, and a novel measure of IPVA perpetration. The study’s longitudinal nature, and the fact the participants were asked to state whether the IPVA took place before or after turning 18, meant that we could capture characteristics of interest both before and at the time the IPVA occurred.

The accuracy of our estimates of association between different potential risk factors and IPVA depends on the accuracy of our measures of these factors and outcome. Most measures used were chosen from a wider range of measures available – for example, we used information about self-harm from two of the three waves where this was available at 10–17 years old. We selected measures based on previous studies using ALSPAC data, that have provided estimates of prevalence for these factors that are in line with those reported in the wider literature ( *Extended data*, Table B). We further accounted for missingness of exposure values through robust multiple imputation methods. In regards to the outcome of IPVA, we parametrised this as broadly as possible (e.g. including emotional abuse and placing the threshold at occurrence ‘ever’), supported by previous work ^7^. Online/digital abuse is increasingly prevalent ^30, 31^, but the IPVA questions did not include any examples of online/digital abuse beyond checking up on someone by phone or text. Therefore, we could not study other common examples, such as sending sexually explicit images. It has also been well documented in the adult literature that IPVA can be under-reported due to recall or reporting biases, particularly perpetration ^32, 33^. Therefore, our estimates of IPVA prevalence are likely to provide a conservative estimate of the true prevalence.

The demographic make-up of those in the ALSPAC cohort limits generalisability of the estimated prevalence of IPVA to relatively affluent, predominantly White UK populations ^16^. Just over one-third of individuals still in the cohort at 21 years old responded to the age 21 wave; internal checks found that those who responded were marginally more likely to be relatively affluent, White, and extremely parentally monitored, and less likely to carry out certain risk-taking behaviours (e.g. use cannabis or other illicit drugs at age 16). Previous work around the effects of participation rates in ALSPAC data, and Norwegian data in young people, found that this phenomenon had a small effect on resulting relative risks and odds ratios for these factors ^29, 34^.

Though we have some information about sexual orientation of the cohort, the IPVA questionnaire did not explicitly ask about the sex of the person who either victimised the individual, or that the person victimised. At least 8% of men and 9% of women had identified as not being 100% heterosexual at age 15 (Extended Data, Table C), but there were 25% and 30% for which this information was not available. Estimated relative risks of not being 100% heterosexual vs. 100% heterosexual, in data where sexual orientation was imputed were very similar to those estimated in complete case data only ( Table 2; Extended Data, Table F). Therefore, estimated effect sizes were negligibly impacted. However, given the potentially large proportions of participants who identified as not being 100% heterosexual, when interpreting sex-specific overall prevalence and relative risks, we cannot assume that victimisation outcomes reported by a male will have been perpetrated by a female, or vice versa.

### Comparison to other literature

We found that most of the risk factors for IPVA victimisation previously identified in north American young people were also potential risk factors for victimisation in a UK cohort ^8– 14^, but this was not the case for low socioeconomic status (SES) ^11, 35^. In the current study there was no clear relationship between area-based deprivation and risks of either victimisation or perpetration (relative risks oscillated when increasing from quintiles 2 to 5, with wide confidence intervals). This is consistent with findings of two recent UK cross-sectional studies (where ethnic minorities were more prevalent and participants were less likely to live with both parents), one suggesting no relationship between SES (as measured on the Family Affluence Scale) and emotional or physical victimisation or perpetration among 11–16 year olds ^7, 36^, the other suggesting no relationship between SES (indicated by weekly spending money) and emotional or online sexual victimisation among 16–19 year olds ^37^. A recent longitudinal study using ALSPAC data estimated that cumulative exposure to low SES (exposure at increasing numbers of time-points; this time SES being dichotomised as quintiles 4–5 vs. 1–3) was associated with a modest increase in risk of IPVA at ages 18–21 (RR=1.4; 95% confidence interval 1.1 to 1.8; i.e. a similar low point estimate to our findings with a narrower confidence interval) ^38^, and IPVA victimisation frequency (a 62% increase in frequency for a one-unit increase in cumulative exposure). Low SES may have a relatively modest relationship with exposure to any IPVA as SES is a very distal factor. This is consistent with analysis based on the Crime Survey for England and Wales ^39^, reporting a stronger association between low SES with more frequent IPVA events. Relative risks of IPVA for individual-level factors (rather than area-level Index of Multiple Deprivation), that are closely related with SES (i.e. education and NEET status), did not provide much clear evidence about the relationship between SES and IPVA, either ( Table 2). Point estimates for the association between high academic achievement and IPVA were negative for victimisation but positive for perpetration. NEET status at 18 years old had a positive association with both victimisation and perpetration, whereas NEET status at 20 years old had a negative association. The relationship between SES and IPVA should still be examined and accounted for in future research. The pathways from different SES indicators to and from both IPVA victimisation and perpetration need to be explored more closely.

We found that most factors studied were risk factors for, as well as victimisation, IPVA perpetration, particularly anxiety, depression, self-harm, anti-social behaviours, cannabis, other illicit drug use, sexual abuse, and witnessing domestic violence – this is a novel addition to the literature given the paucity of reporting of risk factors for perpetration.

### Implications for policy, practice, and research

The fact that a large minority of young men and women aged up to 21 have been victimised and/or perpetrated IPVA, highlights that the focus of primary and secondary prevention of intimate partner violence and abuse needs to be broadened to include this age group. Only relatively recently has there been a sustained UK public health focus on IPVA in young people in particular ^10, 36, 37^. School-based intervention for primary prevention of IPVA (involving information/training provision about identification and reporting to staff, parents and students), that has shown some promise in north America ^40, 41^, is currently being piloted in the UK ^42^. Statistics characterising those at highest risks of exposure to IPVA in this age group, such as those reported in the current study, can inform optimisation of such interventions or future initiatives in similar populations.

Our findings add to the debate around sex differences in violence, and whether the dichotomy of female victimisation and male perpetration widely found in north American adult IPVA studies (including young adults aged 18+, usually college students), similarly applies for UK adolescent and young adults. The prevalence of IPVA victimisation was indeed higher in females compared to males, particularly for physical and sexual victimisation. Nevertheless, the prevalence of victimisation among males was still substantial, at around one in four (compared to around one in three for females). In contrast to the adult literature, we found that the prevalence of perpetration was higher in females than males (about one in four compared to one in five), which was similarly the case when broken down into emotional and physical types, but not sexual, where male perpetration was higher (about one in 63 vs. one in 333). It is possible that the sex differences for relationships of IPVA could differ for this younger age group ^33^. However, it must be noted that these sex differences in prevalence could also be partially driven by sex differences in reporting biases ^33^, and that among those reporting to have been victimised, females were more likely to report negative impact than males, including impacts that would be likely to have long-term health impacts (feeling anxious or depressed, work or studies being affected, drank more alcohol/took more drugs). Elsewhere, we are currently examining the relationship between IPVA and impact in terms of different patterns of types and frequency of IPVA, and whether this might explain any sex differences ^43^. Future qualitative life-course interviews in this age-group will seek to explore in greater depth these differences from the perspectives of young men and young women who have experienced IPVA, including how these experiences have impacted on their lives ^44^. Such interviews provide scope to explore other important factors, such as gender and sexual identity (including choosing not to identify with a sexual orientation), and their experiences, given that IPVA is particularly prevalent for minority groups ^45– 47^.

There is scope for further work in this area to better understand the pathways explaining the associations reported in the current study. We did not include potential risk factors simultaneously in a multivariable regression model as is commonly done in similar epidemiological studies, as our aim here was to identify risk factors and/or characteristics of young people exposed to IPVA and not necessarily to quantify associations whilst ‘adjusting’ for other potential characteristics; such an analysis would likely result in over-adjustment due to the clustering, complex and potentially causal relationships between explanatory variables. For example, it is well known that mental health problems such as depression or anxiety are heavily linked (often bi-directionally) to risky externalizing behaviours such as substance misuse or anti-social behaviour ^48, 49^. Factors identified as being associated with IPVA in this study can be taken forward to be robustly studied within a causal framework, i.e., based on pre-hypothesised pathways to IPVA ^50^.

We found a plethora of factors associated with an increased risk of IPVA. Therefore, our findings provide a focal point for research efforts aimed at elucidating the likely complex pathways to IPVA in young people. Only by understanding such pathways can we improve prevention efforts.

## Data availability

ALSPAC data access is through a system of managed open access. The steps below highlight how to apply for access to ALSPAC data, including access to the Stata/R scripts used for analyses reported in this Research Article.

1. Please read the ALSPAC access policy (PDF, 627kB) which describes the process of accessing the data and samples in detail, and outlines the costs associated with doing so.

2. You may also find it useful to browse our fully searchable research proposals database, which lists all research projects that have been approved since April 2011.

3. Please submit your research proposal for consideration by the ALSPAC Executive Committee. You will receive a response within 10 working days to advise you whether your proposal has been approved.

If you have any questions about accessing data, please email alspac-data@bristol.ac.uk.

The ALSPAC data management plan describes in detail the policy regarding data sharing, which is through a system of managed open access.

### Extended data

Open Science Framework: Risk factors for intimate partner violence and abuse among adolescents and young adults: Extended Data. https://doi.org/10.17605/OSF.IO/K35Y8 ^20^.

The file ‘Extended_data.docx’ contains the following extended data:

- Table A. ALSPAC study questions/responses used to capture romantic relationships.
- Table B. Details about study variables of interest.
- Box A. Notes on imputation.
- Table C. Cohort characteristics.
- Table D. Prevalence of Intimate Partner Violence and Abuse (IPVA) victimisation and perpetration by socio-demographic/ clinical variables and sex.
- Table E. Prevalence of Intimate Partner Violence and Abuse (IPVA) victimisation and perpetration by Adverse Childhood Experiences (ACEs), age at when IPVA occurred, and sex.
- Table F. Relative risks of Intimate Partner Violence and Abuse (IPVA) by 21 years old by socio-demographic/clinical variables and sex (missing risk factor data not imputed – for results where data imputed see Table 2 in main manuscript).

Extended data are available under the terms of the Creative Commons Attribution 4.0 International license (CC-BY 4.0).
