# Supplementary material for: Risk factors for intimate partner violence and abuse among adolescents and young adults: findings from a UK population-based cohort
Source: Wellcome Open Res. 2021 Jan 21;5:176. Originally published 2020 Jul 22. [Version 3] doi: 10.12688/wellcomeopenres.16106.3 (PMC7848855; doi:10.12688/wellcomeopenres.16106.3)
Supplement: Peer review history

# Peer review: Reviewer response for version 3

**DOI:** 10.21956/wellcomeopenres.18247.r42236

**Published:** 2021-01-29

**Authors:** Clare Tanton

**Affiliations:** 1 Global Health and Development, London School of Hygiene and Tropical Medicine, London, UK

I am happy to approve this now. I have no further comments.

Is the work clearly and accurately presented and does it cite the current literature?

Yes

If applicable, is the statistical analysis and its interpretation appropriate?

Partly

Are all the source data underlying the results available to ensure full reproducibility?

Yes

Is the study design appropriate and is the work technically sound?

Partly

Are the conclusions drawn adequately supported by the results?

Yes

Are sufficient details of methods and analysis provided to allow replication by others?

Yes

Reviewer Expertise:

NA

I confirm that I have read this submission and believe that I have an appropriate level of expertise to confirm that it is of an acceptable scientific standard.

# Peer review: Reviewer response for version 3

**DOI:** 10.21956/wellcomeopenres.18247.r42237

**Published:** 2021-01-29

**Authors:** Katharina Goessmann

**Affiliations:** 1 Department of Psychology, Bielefeld University, Bielefeld, Germany

Thank you to the authors for their responses to my comments. I have no additional comments.

Is the work clearly and accurately presented and does it cite the current literature?

Partly

If applicable, is the statistical analysis and its interpretation appropriate?

Yes

Are all the source data underlying the results available to ensure full reproducibility?

Yes

Is the study design appropriate and is the work technically sound?

Yes

Are the conclusions drawn adequately supported by the results?

Partly

Are sufficient details of methods and analysis provided to allow replication by others?

Yes

Reviewer Expertise:

violence, gender-based violence, mental health

I confirm that I have read this submission and believe that I have an appropriate level of expertise to confirm that it is of an acceptable scientific standard.

# Peer review: Reviewer response for version 2

**DOI:** 10.21956/wellcomeopenres.18076.r41421

**Published:** 2020-12-11

**Authors:** Clare Tanton

**Affiliations:** 1 Global Health and Development, London School of Hygiene and Tropical Medicine, London, UK

Thank you for the response to my previous comments and the changes you have made to the manuscript. I have the following additional comments.

 In the response the authors say:

*"Tables 2 and 3 do present relative risks, that is, the prevalence of IPVA at age 0-21 for one level of the factor (e.g. deprivation level 2) / the prevalence of IPVA at age 0-21 for the reference level of the factor (e.g. deprivation level 1)."*

 However, in my understanding of these tables this is not the case. Taking the example the authors give, the relative risk would be 30.3/28.5 for men which is a RR of 1.06, whereas the table shows a RR of 1.09. An OR, however would be ((30.3/69.7)/(28.5/71.5))=1. The difference is less apparent here but for example for the variable sexual identity, the OR is 1.91 but this is not the same as the prevalence almost doubling as the percentages are 40.4 and 26.2. The text needs to represent that these are ORs rather than RRs. 

 Also, in the response the authors say:

*"Original reviewer comment: Text states that "early all factors studied were positively associated with IPVA victimisation by age 21, except for high levels of parental monitoring at age 15, NEET status in young men, and ethnicity minority status in women, which were negatively associated" but I didn't see a negative association for ethnic minority status for women?*

*- In Table 2, the relative risk of IPVA victimisation for ethnicity minority status women (vs. white) was 0.87 (95% CI: 0.56 to 1.36)."*

 Since this OR is close to one and the 95%CI crosses 1 substantially, to me this suggests no evidence for an association between ethnicity and IPVA victimisation.

Is the work clearly and accurately presented and does it cite the current literature?

Yes

If applicable, is the statistical analysis and its interpretation appropriate?

Partly

Are all the source data underlying the results available to ensure full reproducibility?

Yes

Is the study design appropriate and is the work technically sound?

Partly

Are the conclusions drawn adequately supported by the results?

Yes

Are sufficient details of methods and analysis provided to allow replication by others?

Yes

Reviewer Expertise:

Epidemiology, sexual health, violence

I confirm that I have read this submission and believe that I have an appropriate level of expertise to confirm that it is of an acceptable scientific standard, however I have significant reservations, as outlined above.

# Peer review: Reviewer response for version 2

**DOI:** 10.21956/wellcomeopenres.18076.r41477

**Published:** 2020-11-26

**Authors:** Katharina Goessmann

**Affiliations:** 1 Department of Psychology, Bielefeld University, Bielefeld, Germany

I appreciate the opportunity to review this important and high-quality research manuscript. The study provides an interesting descriptive analysis of associations of intimate partner violence (and abuse) victimization and perpetration among UK youth and young adults. The study has several strengths, such as its longitudinal design and large sample. I think the authors addressing of the suggestions from reviewer 1 has already enhanced the manuscript, and I only have some minor comments for further improvement.

- Method, Page 4: Outcome IPVA: IPVA is defined here as violence performed in (ex-)partnerships; however, the instrument used to assess IPVA also includes violence that happened during single occasion intimate encounters (one-night stands). Did you assess whether reported IPVA occurred in a (ex-)relationship or during a one-night stand? Could you please discuss implications of this inclusion?
- The article is about violence performed in (ex-)partnerships, called intimate partner violence (and abuse). The authors use the abbreviation IPVA for this; however, in their table captures, IPVA is used short for “ *interpersonal *violence and abuse”. For reasons of consistency, I suggest to use the term “intimate partner violence and abuse (IPVA)” throughout the manuscript instead of interchanging it with “interpersonal violence”. Plus, the spelling of interpersonal/inter-personal is inconsistent.
- Deprivation is introduced in the methods and tables as a potential risk factor for IPVA, but it is not clear to me what is meant by deprivation which seems to be used here as an SES indicator. Please consider explaining the term “(area-based/level) deprivation” for non-UK readers.
- The authors state that gender (identity) was not included in the analyses due to insufficient data. However, the authors talk about “gender differences” in their discussion of results, even though only sex was included in the analyses. Please make sure to use the correct terminology throughout the manuscript. Also, as sexual and gender minority groups are among those most affected by interpersonal and partner violence, especially SGM youth ﻿(Whitton et al., 2019 ^1^; Dank, Lachman, Zweig, & Yahner, 2014 ^2^; Luo, Stone, & Tharp, 2014 ^3^; Olsen, Vivolo-Kantor, & Kann, 2017 ^4^), I would like to see a deeper discussion of the potential different impacts and associations of sex vs. gender in IPVA.
- Also, the sex/gender of the victimized or perpetrating partners, respectively, was not assessed and information on sexual orientation is missing from 24.5 (of men) and 30.4 % (of women) of the sample. This prevents further interpretations of the results in terms of gender dynamics in partnerships and should be mentioned in the discussion of “gender differences” on page 11. The discussion needs to consider the missing information regarding sex/gender of the relationship partners in this study, as from this data, it cannot be concluded that victimization of a male person means perpetration by a female, and vice versa (except for those participants who indicated to engage only in heterosexual relationships). A more detailed assessment of perpetrator characteristics, relationship types and participants’ sexual orientation is a potential for future study, and should be discussed.
- Wording: I suggest using the term ‘sexual orientation’ instead of ‘sexuality’.
- Discussion, Page 12: As the authors mention, the link between mental health (depression or anxiety etc.) and risky externalizing behaviours such as substance misuse or anti-social behavior is well established; however, the citations provided by the authors (Cho et al. 2014, Pesola et al. 2014) – albeit interesting – are not exactly fitting for this statement in my opinion, as they focus more on the link between substance abuse and anti-social behaviors or depression as a mediating variable between sexual orientation and alcohol abuse. Please consider adding additional references.

Is the work clearly and accurately presented and does it cite the current literature?

Partly

If applicable, is the statistical analysis and its interpretation appropriate?

Yes

Are all the source data underlying the results available to ensure full reproducibility?

Yes

Is the study design appropriate and is the work technically sound?

Yes

Are the conclusions drawn adequately supported by the results?

Partly

Are sufficient details of methods and analysis provided to allow replication by others?

Yes

Reviewer Expertise:

violence, gender-based violence, mental health

I confirm that I have read this submission and believe that I have an appropriate level of expertise to confirm that it is of an acceptable scientific standard, however I have significant reservations, as outlined above.

## References

[1] : Intimate Partner Violence Experiences of Sexual and Gender Minority Adolescents and Young Adults Assigned Female at Birth. *Psychol Women Q*.2019;43(2) : 10.1177/0361684319838972 232-249 10.1177/0361684319838972 31649417PMC6812525

[2] : Dating violence experiences of lesbian, gay, bisexual, and transgender youth. *J Youth Adolesc*.2014;43(5) : 10.1007/s10964-013-9975-8 846-57 10.1007/s10964-013-9975-8 23861097

[3] : Physical dating violence victimization among sexual minority youth. *Am J Public Health*.2014;104(10) : 10.2105/AJPH.2014.302051 e66-73 10.2105/AJPH.2014.302051 25121813PMC4167121

[4] : Physical and Sexual Teen Dating Violence Victimization and Sexual Identity Among U.S. High School Students, 2015. *J Interpers Violence*.2020;35(17-18) : 10.1177/0886260517708757 3581-3600 10.1177/0886260517708757 29294763

# Response

**Published:** 2021-01-07

**Authors:** Annie Herbert

**Affiliations:** University of Bristol, UK

**Katharina Goessmann**, Department of Psychology, Bielefeld University, Bielefeld, Germany 

 I appreciate the opportunity to review this important and high-quality research manuscript. The study provides an interesting descriptive analysis of associations of intimate partner violence (and abuse) victimization and perpetration among UK youth and young adults. The study has several strengths, such as its longitudinal design and large sample. I think the authors addressing of the suggestions from reviewer 1 has already enhanced the manuscript, and I only have some minor comments for further improvement.

*Thank you for your positive response to our work. We really appreciate you taking the time to review our manuscript. We have tried to address your comments point-by-point, below.*

  

1. Method, Page 4: Outcome IPVA: IPVA is defined here as violence performed in (ex-)partnerships; however, the instrument used to assess IPVA also includes violence that happened during single occasion intimate encounters (one-night stands). Did you assess whether reported IPVA occurred in a (ex-)relationship or during a one-night stand? Could you please discuss implications of this inclusion?

*Although the question asking about IPVA experiences states that this can include within either (ex-) relationships or one-night stands, the questionnaire doesn’t ask explicitly about whether the experience took place in one or the other. This was intentional, as relationships for young people (and the young people’s own understandings/interpretations of what it means being in a relationship) are more difficult to categorise than for older adults. In p* *revious research from our group, many young people spoke about more ‘flexible’ encounters where they knew the other person but also had one-night stands with them and were not exclusive (a form of casual relationship which was more common with older teenagers). Although the length/type of a relationship is not necessarily related to the intensity of the relationship experience or to the impact of IPVA on young people, in our qualitative interviews, we use a life-course calendar approach which gives more opportunities to reflect on how these factors might*

*relate to IPVA experiences.* *We have now included the following text in the Methods to make this point.*

*Was:*

*“* *Questions asked about occurrence of eight different examples of emotional, physical, and sexual IPVA victimisation within intimate relationships, including one-night stands (e.g.: ‘Used physical force such as pushing, slapping, hitting or holding you down?’ – physical victimisation).* *”*

*now:*

*“…physical victimisation). The questionnaire did not distinguish between the length/type of relationship (e.g. long-term, one-night stand), as relationships for young people (and the young people’s own understandings/interpretations of what it means being in a relationship) are more difficult to categorise than for older adults.[Reference: Barter et al, NSPCC 2009; Stanley et al, JIPV 2016]”*

 2. The article is about violence performed in (ex-)partnerships, called intimate partner violence (and abuse). The authors use the abbreviation IPVA for this; however, in their table captures, IPVA is used short for “ *interpersonal *violence and abuse”. For reasons of consistency, I suggest to use the term “intimate partner violence and abuse (IPVA)” throughout the manuscript instead of interchanging it with “interpersonal violence”. Plus, the spelling of interpersonal/inter-personal is inconsistent.

*Thank you for spotting this inconsistency. We have now gone through the manuscript and ensured that the updated version only includes IPVA to mean ‘intimate partner violence and abuse’. There should now no longer be any mention of ‘interpersonal’ (or ‘inter-personal’) in the main text or Extended Data.*

 3. Deprivation is introduced in the methods and tables as a potential risk factor for IPVA, but it is not clear to me what is meant by deprivation which seems to be used here as an SES indicator. Please consider explaining the term “(area-based/level) deprivation” for non-UK readers.

*We have now included the following text, which hopefully will make it clearer what we mean by ‘deprivation’ for readers:* *On first mention when describing proportions of young people in relationships,*

**

*was:*

*“…with little difference between those of different deprivation categories…”*

*now:*

*“…with little difference between those of different ‘deprivation’ (socio-economic) categories…”*

*and in two sentences’ time describing variables included in main analyses, was:*

*“These factors were: high area-level deprivation (age 21), ethnic minority status,…”*

*now:*

*“These factors were: high area-level deprivation (i.e. being resident in a geographical area with a high ‘Index of Multiple Deprivation’ score at age 21;[Reference: Lad et al, 2011] an indicator of socio-economic status), ethnicity minority status,…”*

*Given that this is followed by describing a large number of other factors considered in the analysis, more detail/reference is provided in Extended Table B.*

 4. The authors state that gender (identity) was not included in the analyses due to insufficient data. However, the authors talk about “gender differences” in their discussion of results, even though only sex was included in the analyses. Please make sure to use the correct terminology throughout the manuscript. Also, as sexual and gender minority groups are among those most affected by interpersonal and partner violence, especially SGM youth ﻿(Whitton et al., 2019 ^1^; Dank, Lachman, Zweig, & Yahner, 2014 ^2^; Luo, Stone, & Tharp, 2014 ^3^; Olsen, Vivolo-Kantor, & Kann, 2017 ^4^), I would like to see a deeper discussion of the potential different impacts and associations of sex vs. gender in IPVA.

*Thankyou. When we say ‘gender differences’ we do indeed mean ‘sex differences’ and this has now been corrected throughout the updated manuscript. We discuss sex differences in IPVA patterns in the second paragraph of ‘Implications for policy, practice, and research’, and have now expanded this to include gender identity and sexual orientation.*

*Was:*

*“…Elsewhere, we are currently examining the relationship between IPVA and impact in terms of different patterns of sub-types and frequency of IPVA, and whether this might explain any sex differences.* *(40)* *Future qualitative life-course interviews in this age-group will seek to explore in greater depth these differences from the perspectives of young men and young women who have experienced IPVA, including how these experiences have impacted on their lives.”*

*now:*

*“…impacted on their lives. Such interviews provide scope to explore other important factors, such as gender and sexual identity (including choosing not to identify with a sexual orientation), and their experiences, given that IPVA is particularly prevalent for minority groups.[References: Halpern et al, JAH 2004; Dank et al JYA 2014; Peitzmeier AMJPH 2020]”*

 5. Also, the sex/gender of the victimized or perpetrating partners, respectively, was not assessed and information on sexual orientation is missing from 24.5 (of men) and 30.4 % (of women) of the sample. This prevents further interpretations of the results in terms of gender dynamics in partnerships and should be mentioned in the discussion of “gender differences” on page 11. The discussion needs to consider the missing information regarding sex/gender of the relationship partners in this study, as from this data, it cannot be concluded that victimization of a male person means perpetration by a female, and vice versa (except for those participants who indicated to engage only in heterosexual relationships). A more detailed assessment of perpetrator characteristics, relationship types and participants’ sexual orientation is a potential for future study, and should be discussed.

- *While it is true that information on sexual orientation was missing for around one-quarter to one-third of individuals, the main results relate to data that were multiply imputed, including the sexual orientation variable. Estimated relative risks from the multiply imputed datasets were similar to those estimated from data on complete cases only (Extended Data, Table F).*

*It is an important point that for those indicating that they were not 100% heterosexual, we cannot say whether the (ex-)relationships/one-night stands that these IPVA experiences occurred in were opposite- or same-sex pairs, and the sex of the victim or perpetrator that the individual is answering about.*

**

*We have now included the following text in the Discussion under ‘Strengths and Limitations’ to make this point:*

*“* *Though we have some information about sexual orientation of the cohort, the IPVA questionnaire did not explicitly ask about the sex of the person who either victimised the individual, or that the person victimised. At least 8% of men and 9% of women had identified as not being 100% heterosexual at age 15 (Extended Data, Table C), but there were 25% and 30% for which this information was not available. Estimated relative risks of not being 100% heterosexual vs. 100% heterosexual, in data where sexual orientation was multiple imputed were very similar to those estimated in complete case data only (Table 2; Extended Data, Table F). Therefore, estimated effect sizes were negligibly impacted. However, given the potentially large proportions of participants who identify as not being 100% heterosexual, when interpreting sex-specific overall prevalence and relative risks, we cannot assume that victimisation outcomes reported by a male will have been perpetrated by a female, or vice versa.* *”*

*We are currently carrying out more detailed assessment of perpetrator characteristics, relationship types and participants’ sexual orientation, as part of qualitative life-course interviews, which we discuss in the second paragraph of ‘* *Implications for policy, practice, and research’.*

 6. Wording: I suggest using the term ‘sexual orientation’ instead of ‘sexuality’.

*We agree and this has now been done, both in the main manuscript text and Extended Data.*

 7. Discussion, Page 12: As the authors mention, the link between mental health (depression or anxiety etc.) and risky externalizing behaviours such as substance misuse or anti-social behavior is well established; however, the citations provided by the authors (Cho et al. 2014, Pesola et al. 2014) – albeit interesting – are not exactly fitting for this statement in my opinion, as they focus more on the link between substance abuse and anti-social behaviors or depression as a mediating variable between sexual orientation and alcohol abuse. Please consider adding additional references.

*​​​​​​​We have now updated these references to: 1) Wiesner et al, J Abnormal Psych 2003, which directly investigates the bi-directional relationship between depressive symptoms and delinquent behaviour; 2) Campbell et al, Prev Med 2020, which directly investigates the association between different risky externalizing behaviours (including harmful alcohol use, trouble with the police, and problem gambling) and depression and anxiety.*

# Peer review: Reviewer response for version 1

**DOI:** 10.21956/wellcomeopenres.17679.r40128

**Published:** 2020-09-23

**Authors:** Clare Tanton

**Affiliations:** 1 Global Health and Development, London School of Hygiene and Tropical Medicine, London, UK

This is an interesting analysis of IPV data from ALSPAC. It builds on earlier work published in this area from the dataset extending this work to look at risk factors for both IPVA victimisation and perpetration. I have a couple of main comments.

 Firstly, one of the stated strengths of the study is that it is longitudinal and could capture characteristics before and at the time the IPVA occurred. I agree with this, however, I don't think that the analysis and presentation of the results maximises this strength. The main outcome used is experience of IPVA up to age 21 so it is not possible to disentangle the sequence of events for many of these exposures. I wonder whether the authors considered looking at IPVA *after* age 18 and exposures before this time? Then, I wondered whether the results would be better presented by trying to group the exposures by life stage rather than e.g. grouping together mental health at two different ages. Thinking through to implications for prevention and at what age this might occur and how to identify those at risk at different stages of development. Can stronger risk factors be identified earlier in life? I appreciate that the authors say in the discussion that they do not want to construct a multivariable model in this analysis but I think the analysis could be presented in a way that is more coherent with this staged approach to data analysis.

 Secondly, the tables look to present odds ratios rather than relative risks? If I am correct, the text should be adjusted to reflect that these are odds ratios. At the moment it refers in a number of places to a doubling of risk but this is not the case looking at the percentages because the prevalence of IPVA is high.  

 Some additional comments:

**Introduction**

 Could references be added to the following sentence (some from the previous sentence may be moved here):  "Further, most studies of IPVA have been in small or unrepresentative samples, are of adolescents aged under 18 or older adults, are in young girls and women only or investigate risk or protective factors for victimisation but not perpetration."  

**Methods**

*Data*

 Could you add the year the age 21 wave was collected. 

 I found the description of the sample confusing. You state that this is the 3,279 answering questions within the IPVA section, then the next paragraph refers to estimating how many of the cohort had been in a relationship. Was this to define who answered the questions on IPVA?

 The response rate to the survey is not high. Is there a comparison elsewhere between those who responded and those who didn't to better understand the biases that could be referenced. Could you include some further information on this in the discussion.

 There are a lot of missing data. I have presumed that this is due to non-completion of previous surveys (rather than choosing not to answer specific questions) but a table/figure summarising who completed which surveys might be useful within the main paper. I cannot comment on the methods used to deal with the missing data. 

**Results**

 Figure 1 - these results by gender appear in an earlier report of these data where they are compared statistically. There are differences reported by gender for almost all negative impacts - these are larger for the negative impacts which may have a longer-term impact e.g. anxiety, depression, affecting studies. This could be mentioned in the discussion.

 There are a lot of really interesting results presented. I wonder if the tables would be easier to follow if they were split by victimisation and perpetration rather than by gender. This would help align the text since the comparison being made in the text is by gender, rather than by victimisaiton/perpetration.

 Tables  - make number of decimal places consistent for percentages.

 I think tables 2 and 3 would benefit from having the no. people in each of the categories.

 Text states that "early all factors studied were positively associated with IPVA victimisation by age 21, except for high levels of parental monitoring at age 15, NEET status in young men, and ethnicity minority status in women, which were negatively associated" but I didn't see a negative association for ethnic minority status for women?

**Discussion**

 The discussion of gender and violence would benefit from further contextualisation of the results by gender e.g. frequency of occurrence of violence, no. different items experienced/perpetrated, experience of no. different types of violence. The first paragraph of the results could be expanded along these lines. 

 Area level deprivation is a limited measure since it does not tell you anything about an individual's SES. You could also discuss findings from other proxies e.g. education and NEET status in this section.

Is the work clearly and accurately presented and does it cite the current literature?

Yes

If applicable, is the statistical analysis and its interpretation appropriate?

Partly

Are all the source data underlying the results available to ensure full reproducibility?

Yes

Is the study design appropriate and is the work technically sound?

Partly

Are the conclusions drawn adequately supported by the results?

Yes

Are sufficient details of methods and analysis provided to allow replication by others?

Yes

Reviewer Expertise:

Epidemiology, sexual health, violence

I confirm that I have read this submission and believe that I have an appropriate level of expertise to confirm that it is of an acceptable scientific standard, however I have significant reservations, as outlined above.

# Response

**Published:** 2020-11-02

**Authors:** Annie Herbert

**Affiliations:** University of Bristol, UK

Thank you for the time put into reviewing this paper and positive feedback. It has helped us to update and strengthen the paper. We will try to address your comments point by point, below.

*1. Firstly, one of the stated strengths of the study is that it is longitudinal and could capture characteristics before and at the time the IPVA occurred. I agree with this, however, I don't think that the analysis and presentation of the results maximises this strength. The main outcome used is experience of IPVA up to age 21 so it is not possible to disentangle the sequence of events for many of these exposures. I wonder whether the authors considered looking at IPVA after age 18 and exposures before this time?*

 - We did indeed consider the possibility of exploring IPVA at 18-21 only, for the reasons given in the comment. However, we decided that, as the principal purpose of the study was descriptive, and to characterise young relationships in terms of both historical and current sociodemographic, parental behavioural, and clinical factors, that the sequence of events was not a priority, relative to being able to describe the range of factors that we did. For example, hazardous alcohol use and NEET (Not Employed, in Education, or Training) status, factors which have never been described for young people exposed to IPVA in the UK before, were only captured at 18 years or older.

 Further, looking at exposures before turning 18 and outcomes after turning 18 could only interpreted in terms of sequence of events if the sample was further restricted to those who did not report any IPVA at age 0-17. We have started to explore this in other work that looks more deeply at specific variables (e.g. depression) and their relationships with IPVA and attempts to infer causal pathways, but feel it is beyond the scope of the current descriptive study.

 For those interested in descriptive statistics of factors for those exposed to IPVA at age 18-21 in particular (regardless of whether exposed to IPVA at age 0-17 as well), these are provided in Tables D and E of the Extended Data file.

*2. Then, I wondered whether the results would be better presented by trying to group the exposures by life stage rather than e.g. grouping together mental health at two different ages. Thinking through to implications for prevention and at what age this might occur and how to identify those at risk at different stages of development. Can stronger risk factors be identified earlier in life? I appreciate that the authors say in the discussion that they do not want to construct a multivariable model in this analysis but I think the analysis could be presented in a way that is more coherent with this staged approach to data analysis.*

 - As mentioned in the response to comment 1., the purpose of the study is to describe the cohort, rather than disentangle sequences of events. In Table 2, the outcome is IPVA at age 0-21, and so we feel it is more meaningful to group the factors in these tables according to ‘domain’, i.e. demographic, internalising behaviours, etc.  We have now included extra labelling in Table 2 to make this clear. In Table 3, factors all relate to the domain of ‘ACEs’ and the periods when these factors were captured (at age 0-16) are all very similar, and so we did not alter any ordering here.

 However, in Table D of the Extended Data file, the IPVA outcome here is separated out by time (ages 0-17 and 18-21) and so we have re-ordered the factors chronologically as the reviewer suggests.

*3. Secondly, the tables look to present odds ratios rather than relative risks? If I am correct, the text should be adjusted to reflect that these are odds ratios. At the moment it refers in a number of places to a doubling of risk but this is not the case looking at the percentages because the prevalence of IPVA is high.*

 - Tables 2 and 3 do present relative risks, that is, the prevalence of IPVA at age 0-21 for one level of the factor (e.g. deprivation level 2) / the prevalence of IPVA at age 0-21 for the reference level of the factor (e.g. deprivation level 1).

*4. Could references be added to the following sentence (some from the previous sentence may be moved here):  "Further, most studies of IPVA have been in small or unrepresentative samples, are of adolescents aged under 18 or older adults, are in young girls and women only or investigate risk or protective factors for victimisation but not perpetration."*

 - This sentence is supported by the references from the previous sentence (references 8-14).  We will also include them after the current sentence.

*5. Could you add the year the age 21 wave was collected.*

 - Thanks for the suggestion.  In the next version of this manuscript we have updated the following sentence:

 ‘We focussed on participants in the age 21 wave (median and interquartile range [IQR] age 21, 21 to 22); 9,353 were sent a questionnaire (online/paper), to which 3,459 (37%) responded.’

 to:

 ‘We focussed on participants in the age 21 wave (median and interquartile range [IQR] age 21, 21 to 22). All eligible participants who could be contacted (n=9,353) were provided details of an online questionnaire in mid-December 2013, and then sent a series of up to four reminders and three-week intervals, some of these reminders containing a paper version of the same questionnaire. The dataset for the age 21 wave consisted of 3,459/9,353 (37%) who had responded.’

*6. I found the description of the sample confusing. You state that this is the 3,279 answering questions within the IPVA section, then the next paragraph refers to estimating how many of the cohort had been in a relationship. Was this to define who answered the questions on IPVA?*

 - The cohort is indeed all of those who answered questions in the IPVA section, regardless of whether they indicated that they had been in a relationship or not (88% had indicated this, but questions used to capture relationships were on the whole non-specific, and this is likely to be an under-estimate).

 We agree that including the paragraph on relationships within the section on Data is confusing and we apologise. We therefore will move this paragraph into the section ‘Characteristics of the study cohort’ in the next version of the manuscript. Indeed, in this latter section we already describe the proportions who have indicated a relationship by certain ages, so it makes more sense that the text should go here.

*7. The response rate to the survey is not high. Is there a comparison elsewhere between those who responded and those who didn't to better understand the biases that could be referenced. Could you include some further information on this in the discussion.*

 - Thankyou for the suggestion.  We have now extended some of the text that covers external validity and include reference to the description of the full cohort.

 We have changed the following text:

 “The demographic make-up of those that responded to the IPVA questionnaire limits generalisability of the estimated prevalence of IPVA to relatively affluent, predominantly White UK populations. This should not substantially affect the generalisability of reported associations between a range of risk factors and increased risk of IPVA, though it is possible that the magnitude of the association might differ (e.g. be stronger in a more socioeconomically deprived population).”

 to:

 “The demographic make-up of those in the ALSPAC cohort limits generalisability of the estimated prevalence of IPVA to relatively affluent, predominantly White UK populations.(reference: Boyd et al, IJE 2013). Just over one-third of individuals still in the cohort at 21 years old responded to the age 21 wave; internal checks found that those who responded were marginally more likely to be relatively affluent, White, and extremely parentally monitored, and less likely to carry out certain risk-taking behaviours (e.g. use cannabis or other illicit drugs at age 16). Previous work around the effects of participation rates in ALSPAC data, and Norwegian data in young people, found that this phenomenon had a small effect on resulting relative risks and odds ratios for these factors.(references: Howe et al, Epidemiology 2013, Bjertness et al, BMC Public Health 2010)

*8. There are a lot of missing data. I have presumed that this is due to non-completion of previous surveys (rather than choosing not to answer specific questions) but a table/figure summarising who completed which surveys might be useful within the main paper. I cannot comment on the methods used to deal with the missing data.*

 - Extended Data Table C provides the proportions of missing data for all factors, prior to multiple imputation. Exceptions are hospitalisations, risky sexual behaviours, and Adverse Childhood Experiences (ACEs): for these variables it is possible to say if these factors were present, but not if they were absent. However, if we have misunderstood and the comment is asking for other information, please let us know and we’d be happy to try to provide it.

*9. Figure 1 - these results by gender appear in an earlier report of these data where they are compared statistically. There are differences reported by gender for almost all negative impacts - these are larger for the negative impacts which may have a longer-term impact e.g. anxiety, depression, affecting studies. This could be mentioned in the discussion.*

 - Thanks for this suggestion.  We have updated the following sentence:

 “However, it must be noted that these gendered differences in prevalence could also be partially driven by gendered differences in reporting biases, and that among those reporting to have perpetrated IPVA, females were more likely to report negative impact than males.”

 to:

 “However, it must be noted that these gendered differences in prevalence could also be partially driven by gendered differences in reporting biases, and that among those reporting to have been victimised, females were more likely to report negative impact than males, including impacts that would be likely to have long-term health impacts (feeling anxious or depressed, work or studies being affected, drank more alcohol/took more drugs). We are currently examining the relationship between IPVA and impact in terms of different patterns of sub-types and frequency of IPVA, and whether this might explain any gender differences.(reference: Herbert et al, unpublished)”

*10. There are a lot of really interesting results presented. I wonder if the tables would be easier to follow if they were split by victimisation and perpetration rather than by gender. This would help align the text since the comparison being made in the text is by gender, rather than by victimisaiton/perpetration.*

 - Thanks for this suggestion, which we agree will make interpretation easier. We have now updated Tables 2 and 3, and Extended Data Tables D, E, and F, as such.

*11. Tables  - make number of decimal places consistent for percentages.*

 - Apologies, this has now been done.

*12. I think tables 2 and 3 would benefit from having the no. people in each of the categories.*

 - Tables 2 and 3 are of pooled results from multiply imputed data.  We have now included footnotes to make this clearer. Numbers of people in each of the categories in the raw data (including number of those missing) are provided in Extended Data Table C.

*13. Text states that "early all factors studied were positively associated with IPVA victimisation by age 21, except for high levels of parental monitoring at age 15, NEET status in young men, and ethnicity minority status in women, which were negatively associated" but I didn't see a negative association for ethnic minority status for women?*

 - In Table 2, the relative risk of IPVA victimisation for ethnicity minority status women (vs. white) was 0.87 (95% CI: 0.56 to 1.36).

*14. The discussion of gender and violence would benefit from further contextualisation of the results by gender e.g. frequency of occurrence of violence, no. different items experienced/perpetrated, experience of no. different types of violence. The first paragraph of the results could be expanded along these lines.*

 - In Table 1, we cover the prevalence of different subtypes of IPVA victimisation and perpetration; these results, and the comparison between genders, are discussed in the first paragraph of the Results section, under ‘Prevalence and impact of IPVA victimisation and perpetration’. Our analysis and results do not touch on other dimensions of IPVA such as frequency of occurrence (we defined IPVA as having endorsed at least ‘Once’ for reasons described in the section ‘Outcome: IPVA’), or number of items experienced/perpetrated. We are exploring the latter dimensions in a separate study, which is in progress.

*15. Area level deprivation is a limited measure since it does not tell you anything about an individual's SES. You could also discuss findings from other proxies e.g. education and NEET status in this section.*

 - We appreciate that point that education and NEET status are individual-level factors that could tell us something about the relationship between SES and IPVA. In the next version of the manuscript, we have updated the paragraph about SES to include the following text:

 “Relative risks of IPVA for individual-level factors (rather than area-level Index of Multiple Deprivation), that are closely related with SES (i.e. education and NEET status), did not provide clear evidence about the relationship between SES and IPVA, either (Table 2). High academic achievement had negligible associations with IPVA for men and appeared to have negative associations with IPVA for women. NEET status at 18 years old had a positive association with both victimisation perpetration, whereas NEET status at 20 years old had a negative association with IPVA victimisation and positive association with perpetration. The relationship between SES and IPVA should still be examined and accounted for in future research. The pathways from different SES indicators to and from both IPVA victimisation and perpetration need to be explored more closely.”

Additionally:

 Whilst updating the manuscript in response to the reviewer comments, we realised that the exacting wording of the question around impact related only to victimisation. Therefore, we have also updated Figure 1 to only include impact outcomes relative to victimisation status, not victimisation and perpetration.

# Response

**Published:** 2021-01-07

**Authors:** Annie Herbert

**Affiliations:** University of Bristol, UK

Thank you for the response to my previous comments and the changes you have made to the manuscript.

**Thanks Clare, for taking the time to look over the updated version.  We have tried to address your outstanding comments, below.**

 1. In the response the authors say:

*"Tables 2 and 3 do present relative risks, that is, the prevalence of IPVA at age 0-21 for one level of the factor (e.g. deprivation level 2) / the prevalence of IPVA at age 0-21 for the reference level of the factor (e.g. deprivation level 1)."*

 However, in my understanding of these tables this is not the case. Taking the example the authors give, the relative risk would be 30.3/28.5 for men which is a RR of 1.06, whereas the table shows a RR of 1.09. An OR, however would be ((30.3/69.7)/(28.5/71.5))=1. The difference is less apparent here but for example for the variable sexual identity, the OR is 1.91 but this is not the same as the prevalence almost doubling as the percentages are 40.4 and 26.2. The text needs to represent that these are ORs rather than RRs.

**Having gone back to the original R code used, you’re absolutely right and indeed, we pooled results of logistic regression models across imputed datasets.  Thanks for spotting this, and we apologise for the error.  We have updated the results to reported estimated RRs (as per Zhang *et al*, JAMA 1998).  These changes affect Tables 2 and 3, and Table F in Extended Data.  The (RR) estimates have moved closer to the null compared with the original (OR) estimates, but general conclusions about the strongest risk/protective factors have remained unchanged.**

 2. Also, in the response the authors say:

*"Original reviewer comment: Text states that "early all factors studied were positively associated with IPVA victimisation by age 21, except for high levels of parental monitoring at age 15, NEET status in young men, and ethnicity minority status in women, which were negatively associated" but I didn't see a negative association for ethnic minority status for women?*

*- In Table 2, the relative risk of IPVA victimisation for ethnicity minority status women (vs. white) was 0.87 (95% CI: 0.56 to 1.36)."*

 Since this OR is close to one and the 95%CI crosses 1 substantially, to me this suggests no evidence for an association between ethnicity and IPVA victimisation.

**We take your point that the confidence interval includes a null association, and (given the updated results to report RRs not ORs), the updated point estimate for ethnic minority status women is now not below 1. Therefore, we have removed this text in the updated manuscript.**
